# Horizontal Gene Transfer Clarifies Taxonomic Confusion and Promotes the Genetic Diversity and Pathogenicity of Plesiomonas shigelloides

**DOI:** 10.1128/mSystems.00448-20

**Published:** 2020-09-15

**Authors:** Zhiqiu Yin, Si Zhang, Yi Wei, Meng Wang, Shuangshuang Ma, Shuang Yang, Jingting Wang, Chao Yuan, Lingyan Jiang, Yuhui Du

**Affiliations:** a Key Laboratory of Molecular Microbiology and Technology of the Ministry of Education, TEDA College, Nankai University, Tianjin, People’s Republic of China; b TEDA Institute of Biological Sciences and Biotechnology, Nankai University, Tianjin, People’s Republic of China; c Tianjin Key Laboratory of Microbial Functional Genomics, TEDA College, Nankai University, Tianjin, People’s Republic of China; d Key Laboratory of Molecular Medicine and Biotherapy, School of Life Sciences, Beijing Institute of Technology, Beijing, People’s Republic of China; e Department of Clinical Laboratory, Tianjin First Central Hospital, Tianjin, People’s Republic of China; ExxonMobil Research and Engineering

**Keywords:** *Plesiomonas shigelloides*, comparative genomics, horizontal gene transfer, pan-genome, pathogenesis

## Abstract

Plesiomonas shigelloides is an emerging pathogen that has been shown to be involved in gastrointestinal diseases and extraintestinal infections in humans. However, the taxonomic position, evolutionary dynamics, and pathogenesis of P. shigelloides remain unclear. We reported the draft genome sequences of 12 P. shigelloides strains representing different serogroups. We were able to determine a clear distinction between P. shigelloides and other members of *Enterobacterales* via core genome phylogeny, Neighbor-Net network, and average genome identity analysis. The pan-genome analysis of P. shigelloides revealed extensive genetic diversity and presented large flexible gene repertoires, while the core genome phylogeny exhibited a low level of clonality. The discordance between the core genome phylogeny and the pan-genome phylogeny indicated that flexible accessory genomes account for an important proportion of the evolution of P. shigelloides, which was subsequently characterized by determinations of hundreds of horizontally transferred genes (horizontal genes), massive gene expansions and contractions, and diverse mobile genetic elements (MGEs). The apparently high levels of horizontal gene transfer (HGT) in P. shigelloides were conferred from bacteria with novel properties from other taxa (mainly *Vibrionaceae* and *Aeromonadaceae*), which caused the historical taxonomic confusion and shaped the virulence gene pools. Furthermore, P. shigelloides genomes contain many macromolecular secretion system genes, virulence factor genes, and resistance genes, indicating its potential to cause intestinal and invasive infections. Collectively, our work provides insights into the phylogenetic position, evolutionary dynamic, and pathogenesis of P. shigelloides at the genomic level, which could facilitate the observation and research of this important pathogen.

**IMPORTANCE** The taxonomic position of P. shigelloides has been the subject of debate for a long time, and until now, the evolutionary dynamics and pathogenesis of P. shigelloides were unclear. In this study, pan-genome analysis indicated extensive genetic diversity and the presence of large and variable gene repertoires. Our results revealed that horizontal gene transfer was the focal driving force for the genetic diversity of the P. shigelloides pan-genome and might have contributed to the emergence of novel properties. *Vibrionaceae* and *Aeromonadaceae* were found to be the predominant donor taxa for horizontal genes, which might have caused the taxonomic confusion historically. Comparative genomic analysis revealed the potential of P. shigelloides to cause intestinal and invasive diseases. Our results could advance the understanding of the evolution and pathogenesis of P. shigelloides, particularly in elucidating the role of horizontal gene transfer and investigating virulence-related elements.

## Abstract (precis)

The taxonomic position of P. shigelloides has been the subject of debate for a long time, and until now, the evolutionary dynamics and pathogenesis of P. shigelloides were unclear. In this study, pan-genome analysis indicated extensive genetic diversity and the presence of large and variable gene repertoires. Our results revealed that horizontal gene transfer was the focal driving force for the genetic diversity of the P. shigelloides pan-genome and might have contributed to the emergence of novel properties. *Vibrionaceae* and *Aeromonadaceae* were found to be the predominant donor taxa for horizontal genes, which might have caused the taxonomic confusion historically. Comparative genomic analysis revealed the potential of P. shigelloides to cause intestinal and invasive diseases. Our results could advance the understanding of the evolution and pathogenesis of P. shigelloides, particularly in elucidating the role of horizontal gene transfer and investigating virulence-related elements.

## INTRODUCTION

The genus *Plesiomonas* used to be classified into the family *Vibrionaceae* many years ago and is currently classified as a member of the family *Enterobacteriaceae* (1). Plesiomonas shigelloides, represented as a single species in the genus *Plesiomonas*, is a Gram-negative, flagellated, facultative anaerobic, rod-shaped bacterium. It is widespread in aquatic environments and is also found in a wide range of hosts, including humans, dogs, fish, sheep, cows, pigs, and turkey vultures (2). A serotyping scheme using antigenic variation in the lipopolysaccharide (O-antigen) and flagella (H-antigen) has been used for classification, identification, and epidemiological investigation of P. shigelloides. At present, 102 O-antigens and 51 H-antigens have been recognized (3).

The taxonomic position of P. shigelloides has long been debated. It originally was designated a member of group C27, together with the major antigen Shigella sonnei phase I. Subsequently, P. shigelloides was found to possess a number of properties in common with other bacterial taxa, which caused it to be misclassified into the genus *Aeromonas*, or family *Vibrionaceae* (4, 5). Presently, phylogenetic analysis based on 5S and 16S data and on data from multilocus sequence typing (MLST) indicated that this taxon is rooted within *Enterobacteriaceae*, but not within the *Proteus* clade (6–9). The genetic diversity of this bacterium has been verified by pulsed-field gel electrophoresis (PFGE), random amplified polymorphic DNA (RAPD) typing, and MLST (2, 9, 10). However, researchers who used the previously available methods encountered difficulty in obtaining high-resolution taxonomy as well as detailed information on the genetic diversity and phylogenetic relationships of P. shigelloides. Accurate taxonomy can improve understanding of the evolution, epidemiology, and pathogenicity of bacteria. However, the phylogenetic position and genetic diversity of P. shigelloides have not yet been understood from the whole-genome perspective. Additionally, the ubiquitous living environment of P. shigelloides and the high density of cohabitating bacteria in the host niche are conducive for genetic exchange between donor and recipient strains (9). Horizontal gene transfer (HGT) mediates DNA transmission, giving rise to novel properties associated with niche adaptation and pathogenicity (11, 12). However, the role played by HGT in the pan-genome of P. shigelloides is also not comprehensively understood.

P. shigelloides has been found to be associated with gastroenteritis and diarrhea in humans, including acute secretory gastroenteritis, invasive shigellosis-like diseases, and cholera-like diseases (1). Outbreaks are generally associated with seafood/fish consumption or contaminated water. Notably, it also has been reported to cause severe extraintestinal diseases, including bacteremia, peritonitis, pneumonia, meningitis, hepatobiliary disease, septicemia, and pseudoappendicitis (13). P. shigelloides possesses cytotoxicity and enterotoxicity and can adhere to and invade host cells *in vitro* (14). Excluding ampicillin, this bacterium was found to be susceptible to a variety of antibiotics (15, 16); however, multidrug-resistant traits have also been reported (17). Although clinical and epidemiological data already imply a role of P. shigelloides in human infections, the underlying mechanisms of pathogenicity and the molecular evolution of this bacterium have not yet been comprehensively investigated.

To better understand the genetic diversity and pathogenic potential of P. shigelloides, we generated draft genome sequences for 12 strains representing different serogroups whose O-antigen gene clusters have been reported previously (18). Phylogeny was performed for a larger collection of the members of *Enterobacterales*, focusing on the phylogenetic position of P. shigelloides and species-specific genetic properties. The pan-genome analysis was constructed to characterize the genetic diversity and evolutionary dynamics of P. shigelloides. We also detected potential horizontally transferred genes (horizontal genes) with their donor taxa, gene expansions and contractions in the evolution, and mobile genetic elements (MGEs). The comparative genome analysis was performed to characterize virulence-related genetic elements (macromolecular secretion systems [SSs] and virulence factors). The resistance genes were identified, and antimicrobial susceptibility profiles were generated for 18 antibiotics, including aminoglycosides, beta-lactams, sulfonamides, fluoroquinolones, tetracyclines, and polymyxin.

## RESULTS AND DISCUSSION

### The phylogenetic position of P. shigelloides in the order *Enterobacterales*.

**FIG 1:**
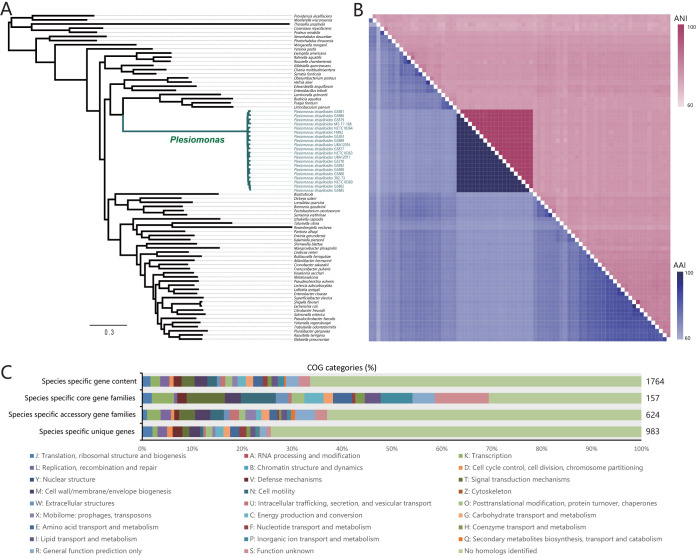
Phylogenetic analysis and whole-genome nucleotide and amino acid identities. (A) ML phylogeny was constructed based on SNPs across 307 single-copy core gene families shared by the 20 P. shigelloides genomes and 59 reference genomes of other *Enterobacterales*. (B) The heat map presents average nucleotide identities (red upper section of matrix) and amino acid identities (blue lower section of matrix). (C) Distribution of COG categories for each set of species-specific gene repertoires.

Currently, P. shigelloides is classified into the family *Enterobacteriaceae* (unclassified *Enterobacterales* in NCBI taxonomy database) after having been classified for many years in the genus *Aeromonas*, family *Vibrionaceae*, or the tribe *Proteeae* (1). We generated a high-resolution phylogeny based on 307 single-copy core gene families from 79 genomes to evaluate the phylogenetic position of P. shigelloides in the order *Enterobacterales*; these consisted of 20 P. shigelloides genomes (12 newly sequenced and 8 publicly available genomes) and 59 other genus/species reference genomes (see Table S1 in the supplemental material). As shown in Fig. 1A, all of the P. shigelloides strains formed a monophyletic clade with a long branch length and were deeply nested within *Enterobacteriaceae*, suggesting divergence between P. shigelloides and other members of *Enterobacterales*. The separate clade of P. shigelloides diverged early in the phylogeny, showing a clear distinction between P. shigelloides and other enterobacteria (Fig. 1A; see also Fig. S1A in the supplemental material). We also constructed a neighbor-joining (NJ) phylogenetic split tree network (Neighbor-Net network) to better visualize the relationships between these enterobacteria. As shown in Fig. S1, the *Enterobacterales* strains were resolved into eight groups: *Enterobacteriaceae*, *Erwiniaceae*, *Pectobacteriaceae*, *Morganellaceae*, *Yersiniaceae*, *Hafnia*, *Budviciaceae*, and *Plesiomonas*. P. shigelloides represented a separate group (*Plesiomonas* group) distinct from the other enterobacterial groups. Some authors previously suggested that P. shigelloides should be classified into the tribe *Proteeae* (*Proteus*, *Morganella*, *Providencia*) (6); however, as indicated in Fig. 1A and Fig. S1, P. shigelloides is not closely related to Proteus mirabilis (*Morganellaceae* group). The nearest neighboring group to P. shigelloides is *Budviciaceae*, which comprises Budvicia aquatica, Pragia fontium, Leminorella grimontii, and Limnobaculum parvum.

10.1128/mSystems.00448-20.1 FIG S1 Core genome phylogeny. (A) Radial tree layout of the core genome tree (Fig. 1A). The subgroup of *Enterobacterales* was completed according to the NCBI taxonomy database. Different groups are shown in different colors. (B) The Neighbor-Net network based on an uncorrected p-distance transformation inferred from the 307 single-copy core gene families shared by the 79 *Enterobacterales* strains. Download FIG S1, EPS file, 2.5 MB. Copyright © 2020 Yin et al. 2020 Yin et al. This content is distributed under the terms of the Creative Commons Attribution 4.0 International license.

10.1128/mSystems.00448-20.4 TABLE S1 Genetic characteristics of the strains in the current study. Download Table S1, XLSX file, 0.02 MB. Copyright © 2020 Yin et al. 2020 Yin et al. This content is distributed under the terms of the Creative Commons Attribution 4.0 International license.

The average nucleotide identity (ANI) and average amino acid identity (AAI) were also used to measure the genetic relatedness between these enterobacteria. The ANI and AAI values determined from comparisons between P. shigelloides and other enterobacteria were 69.2% to 77.1% and 57.8% to 67.5%, respectively, exhibiting low levels of relatedness between P. shigelloides and other enterobacteria. The strains within the *Plesiomonas* group showed ANI and AAI values higher than 96.6% and 97.8%, respectively, which exceeded the recommended 95% threshold value for species circumscription (19, 20).

### Species-specific gene repertoires.

We also characterized the P. shigelloides-specific gene repertoire, which consisted of gene families that were present in P. shigelloides genomes and absent in other 59 enterobacterial genomes. This repertoire was comprised of a species-specific core (genes shared among all 20 genomes), accessory gene families (genes shared between at least 2 genomes and among fewer than 20 genomes), and unique gene families (genes that existed in only 1 genome). Functions of each gene set were characterized by analysis using the Cluster of Orthologous Group (COG). A total of 1,764 gene families were identified and made up the species-specific gene repertoires (Table S2); among them, 157 (8.9%) were species-specific core gene families. These core gene families were enriched with those involved in information storage and processing (“K: transcription” [7 genes]), cellular processes and signaling (“T: signal transduction mechanisms” [12 genes] and “N: cell motility” [11 genes]), and metabolism (“C: energy production and conversion” [6 genes], “E: amino acid transport and metabolism” [6 genes], and “P: inorganic ion transport and metabolism” [10 genes]) (Fig. 1C). We then used the KEGG annotation to assign the functional category of the species-specific core gene families: 54 (34.4%) gene families had prominent KEGG annotation and were assigned to “energy metabolism” (4 genes), “signal transduction” (7 genes), “cellular community” (4 genes), and “cell motility” (5 genes) (Fig. S2A). These gene families (retained in a species-specific fashion) mainly participated in nutrition metabolism and signal transduction, which may confer to P. shigelloides distinct nutritional and genetic properties from other members of *Enterobacterales*. The results suggest that P. shigelloides has the potential to survive in specific nutritional environments and to respond to rapidly changing environments. Considering that P. shigelloides has a worldwide distribution, these species-specific core gene families may play an important role in environmental adaptation and competitiveness.

10.1128/mSystems.00448-20.2 FIG S2 (A) Function enrichment of the species-specific core gene families and the core gene families acquired by HGT based on the KEGG annotation. (B) The distribution of COG categories for island genes in P. shigelloides. (C) The distribution of COG categories for island genes in each genome. Download FIG S2, EPS file, 1.9 MB. Copyright © 2020 Yin et al. 2020 Yin et al. This content is distributed under the terms of the Creative Commons Attribution 4.0 International license.

10.1128/mSystems.00448-20.5 TABLE S2 List of specific gene families identified in P. shigelloides, including core, accessory, and strain-specific gene families. Download Table S2, XLSX file, 0.1 MB. Copyright © 2020 Yin et al. 2020 Yin et al. This content is distributed under the terms of the Creative Commons Attribution 4.0 International license.

The remaining species-specific gene repertoires comprised of 624 species-specific accessory gene families and 983 unique species-specific genes (Fig. 1C). High proportions of both species-specific accessory gene families (69.4%) and unique genes (76.4%) were poorly characterized (“R: general function prediction only” [25 accessory genes and 13 unique genes], “S: function unknown” [15 accessory genes and 8 unique genes], and “no homologs identified” [393 accessory genes and 730 unique genes]). The remaining 30.6% of the species-specific accessory gene families were prominently enriched in “K: transcription” (17 genes), “T: signal transduction mechanisms” (20 genes), “M: cell wall/membrane/envelope biogenesis” (19 genes), and “N: cell motility” (17 genes). The remaining 23.6% species-specific unique gene families were prominently enriched in “J: translation, ribosomal structure and biogenesis” (20 genes), “L: replication, recombination, and repair” (19 genes), “V: defense mechanisms” (19 genes), “M: cell wall/membrane/envelope biogenesis” (21 genes), and “E: amino acid transport and metabolism” (18 genes). These genes conferred various functions to P. shigelloides members to enable adaptation to diverse niches. In addition, the high proportion of unknown functional genes in the species-specific gene repertoires requires further research.

### Core and pan-genome analysis of P. shigelloides revealed extensive genetic diversity.

**FIG 2:**
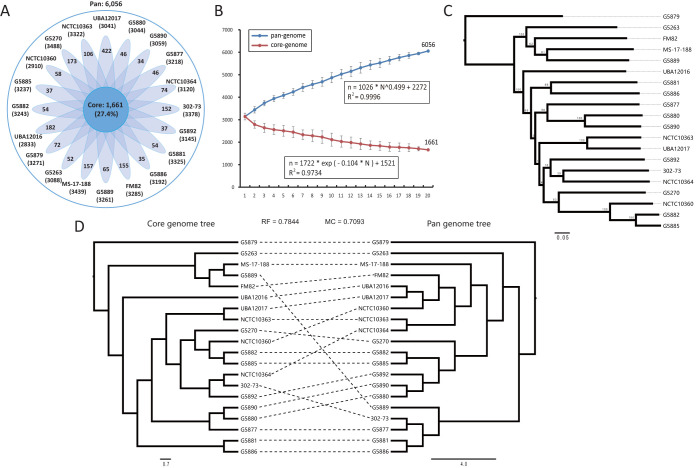
Genetic diversity of genome in P. shigelloides. (A) Strain-specific gene families of 20 P. shigelloides genomes. The number of core gene families shared by all strains is shown in the center. (B) Core and pan-genome curves showing the downward trend of the core gene families and the upward trend of the pan-gene families with the increase in the number of genomes. The error bars indicate standard deviations of the number of core and pan-gene families. The deduced mathematical functions of the core and pan-genome curves are also reported. (C) ML phylogeny constructed based on SNPs across 1,522 single-copy core gene families shared by the 20 P. shigelloides genomes. The interior node values shown in the tree represent bootstrap values (100 replicates). (D) Comparison between the core genome tree and pan-genome tree. Normalized Robinson-Foulds (nRF) and normalized matching cluster (nMC) scores were used to measure the congruence of the two trees.

**FIG 3:**
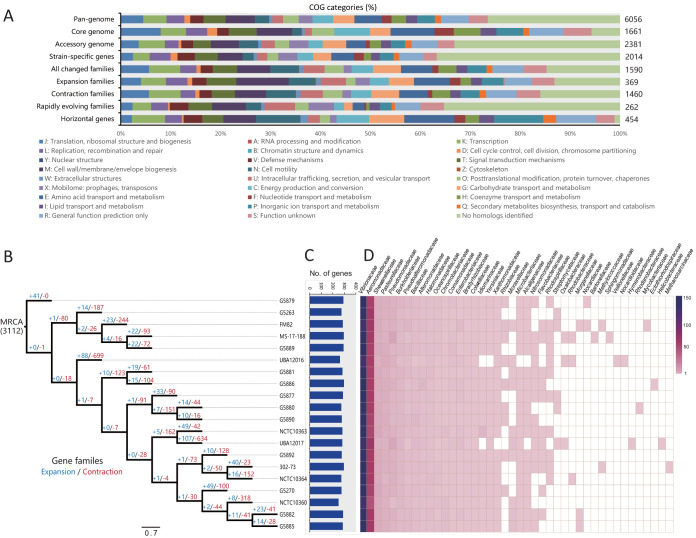
Functional categories and evolutionary dynamics of gene families. (A) Distribution of COG categories for each gene family set. (B) Expansion and contraction of gene families in each branch of the core genome tree. The number of expanded genes (+; blue) and the number of contracted genes (−; red) are shown in each branch. (C) Distribution of horizontal genes in P. shigelloides genomes. (D) The potential donor bacterial taxa providing donor genes for HGT.

We characterized the core and pan-genome among 20 P. shigelloides strains to assess their genetic diversity. A total of 6,056 pan-genome gene families were identified (Fig. 2A; see also Table S3). The core gene families (1,661, 27.4%) were enriched in “J: translation, ribosomal structure and biogenesis” (148, 8.9%), “M: cell wall/membrane/envelope biogenesis” (132, 7.9%), “C: energy production and conversion” (132, 7.9%), and “E: amino acid transport and metabolism” (163, 9.8%) (Fig. 3A). The variable genetic content made up a high proportion (72.6%) of the pan-genome, indicating a high degree of genetic variation in P. shigelloides. The variable genetic content is an important source of evolutionary novelty that facilitates rapid adaptation via HGT (11, 21). The accessory gene families (2,381, 39.3%) were enriched in “K: transcription” (152 genes), “M: cell wall/membrane/envelope biogenesis” (164 genes), and “G: carbohydrate transport and metabolism” (137 genes). Additionally, 2,014 strain-specific gene families (33.3%) were present in only one genome, suggesting a high frequency of horizontal gene acquisition from other bacterial taxa (22). The distribution of these strain-specific genes in P. shigelloides was diverse, ranging from 34 genes (strain G5890) to 422 genes (strain UBA12017), indicating high genomic plasticity (Fig. 2A). These unique genes carried diverse functions, such as “L: replication, recombination, and repair” (78 genes), “T: signal transduction mechanisms” (86 genes), “M: cell wall/membrane/envelope biogenesis” (93 genes), “X: mobilome: prophages, transposons” (72 genes), and “E: amino acid transport and metabolism” (85 genes) (Fig. 3A).

10.1128/mSystems.00448-20.6 TABLE S3 List representing the pan-genome of P. shigelloides, including core, accessory, and strain-specific gene families. Download Table S3, XLSX file, 0.3 MB. Copyright © 2020 Yin et al. 2020 Yin et al. This content is distributed under the terms of the Creative Commons Attribution 4.0 International license.

We further constructed the core and pan-genome curves (Fig. 2B). The sizes of the core gene families decreased continuously as the number of genomes increased, indicating that the core gene families would eventually reach a stable minimum. We estimated this minimum number (*N* = 1,521) by the use of a mathematical model that fitted a single exponential decay function (23). The pan-genome showed a clear linear upward trend conforming to Heap’s law for the pan-genome model (24). The pan-genome model with the positive exponent *γ* = 0.499 (*γ* > 0) exhibited an open pan-genome, suggesting that P. shigelloides has a large source of gene pools and has great potential to acquire novel genes.

### Core and pan-genome phylogenetic analysis.

To assess the phylogenetic relationships among P. shigelloides strains, we constructed a core genome tree using the concatenated nucleotide sequences of 1,522 single-copy core gene families shared by all 20 strains. Our genome phylogeny appears to have many deep clades and very few major clusters (Fig. 2C), exhibiting extensive genetic diversity and a low level of clonality of P. shigelloides strains. Similarly, Vibrio cholerae and Vibrio parahaemolyticus also have extensive genetic diversity and high recombination rates, resulting to a relatively low level of clonality (25, 26). Thus, the low level of clonality revealed by the core genome tree indicated a high level of recombination in the core genome of P. shigelloides.

We also constructed a pan-genome tree and compared the core tree and pan-genome tree to quantify the correlation between phylogeny and genome composition. As shown in Fig. 2D, there was discordance in the topology of the branching order and phylogenetic placement between the two trees. We calculated normalized Robinson-Foulds (nRF) and normalized matching-cluster (nMC) values to evaluate the topological correlation between the core and pan-genome tree. The scores of both nRF and nMC ranged from 0 to 1, indicating congruence or noncongruence between the two trees. In this study, the nRF and nMC scores were 0.7844 and 0.7093, respectively, indicating that the relationships among the parts of the pan-genome exhibited low congruence to the phylogenetic relationships of the core gene families. This result may have been due to the presence of a large number of accessory genomes. Our study data suggested that these variable gene families might play an important role in phylogenetic relationships and that genetic diversity has been of great significance in the evolution of P. shigelloides.

### Gene gain and loss for the P. shigelloides pan-genome.

Gene gain and loss during evolution can increase the environmental adaptability and competitiveness of bacteria (27, 28). We identified gene family expansion and contraction at each branch of core genome phylogeny to explore the dynamics of gene families over the evolution of the species. A total of 3,112 gene families were predicted to represent gene repertoires of the most recent common ancestor (MRCA) of the 20 P. shigelloides genomes. A high proportion (1,590, 51.1%) was identified as representing changes in families throughout the phylogeny (Fig. 3B), suggesting the variety and plasticity of these gene repertoires. The evolutionary dynamics were indicated by gene expansion and contraction determinations on each branch (Fig. 3B). The number of changed families on each branch showed variation, and the changes in the family occurred at higher levels in the external branches than in the internal branch. Additionally, the number of families showing contraction (1,460 gene families) was greater than that of the families showing expansion (369 gene families), which was generally shown in separate branches. Many pathogens have undergone massive gene loss as an adaptive response to living within hosts (27). The trend toward massive gene loss suggested that gene loss might play an important role in the adaptive evolution of P. shigelloides. Gene gain and loss are also associated with functional change (29). The contracted families were enriched in “K: transcription” (104, 7.1%), “M: cell wall/membrane/envelope biogenesis” (102, 7.0%), “N: cell motility” (97, 6.6%), and “E: amino acid transport and metabolism” (94, 6.4%). The expanded families were enriched in “M: cell wall/membrane/envelope biogenesis” (44, 11.9%) and “E: amino acid transport and metabolism” (30, 8.1%). Genes assigned to “M: cell wall/membrane/envelope biogenesis” and “E: amino acid transport and metabolism” were prominently represented in both families, suggesting that environmental changes may be contributors to their adaptive evolution as a consequence of requiring alterations in the cell wall and metabolism. Our results revealed massive changes in gene families which shaped the functional divergence among the P. shigelloides strains.

### The taxonomic confusion regarding P. shigelloides due to hundreds of horizontal genes.

HGT is the major driver of genetic diversity and speciation of bacteria (11). The acquisition of foreign genes could confer new properties, which are crucial for pathogenicity and adaptation into diverse niches (11, 12). Here, we examined the potential horizontal genes in P. shigelloides genomes and tracked the potential donor taxa. A total of 454 potential horizontal gene families were identified (Fig. 3A; see also Table S4), with an average genome containing 281.9 ± 11.5 horizontal genes (Fig. 3C). These horizontal genes were mainly involved in “N: cell motility” (48 genes, 10.6%), “G: carbohydrate transport and metabolism” (38, 8.4%), “E: amino acid transport and metabolism” (54, 11.9%), and “P: inorganic ion transport and metabolism” (53, 11.7%) (Fig. 3A), indicating that these foreign genes might confer adaptations of P. shigelloides to diverse niches. In addition, a total of 42 potential donor taxa were identified (Fig. 3D). The families *Vibrionaceae* and *Aeromonadaceae* appeared to be the main donor taxa. On average, one P. shigelloides genome acquired 142.8 ± 8.5 and 67.1 ± 4.0 horizontal genes from *Vibrionaceae* and *Aeromonadaceae*, respectively. These results indicated that P. shigelloides shares some properties with members of both *Vibrionaceae* and *Aeromonadaceae.* This observation is not that surprising since aquatic environments are preferred by all *Vibrionaceae*, *Aeromonadaceae*, and P. shigelloides, which provides many opportunities for recombination.

10.1128/mSystems.00448-20.7 TABLE S4 Summary of horizontal genes identified in P. shigelloides. Download Table S4, XLSX file, 0.2 MB. Copyright © 2020 Yin et al. 2020 Yin et al. This content is distributed under the terms of the Creative Commons Attribution 4.0 International license.

HGT also contributed to the core genome of P. shigelloides. A total of 130 core gene families can potentially be acquired via HGT, including 64 from *Vibrionaceae* and 35 from *Aeromonadaceae* (Table S5). These horizontal core gene families contributed to the special genomic content, which conferred to P. shigelloides a number of special properties distinct from those of other members of *Enterobacteriaceae*. Historically, the confusion concerning P. shigelloides taxonomy had been focused on the genus *Aeromonas*, family *Vibrionaceae*, mainly because P. shigelloides possesses a number of biochemical properties in common with these bacteria, including cytochrome oxidases, fermentative metabolism, ecologic associations, and disease presentations (5). Based on KEGG annotation, 104 (80.0%) horizontal core gene families were prominently assigned to “carbohydrate metabolism” (13 genes), “energy metabolism” (15 genes), “amino acid metabolism” (13 genes), “membrane transport” (13 genes), and “signal transduction” (13 genes) (Fig. S2A). Several pathway modules were present in the horizontal core gene families (Table S5), for example, a cytochrome *bc*_1_ complex (*Vibrionaceae*) involved in oxidative phosphorylation, *afuABC* (*Vibrionaceae*) encoding an iron III ABC transporter, and *pstABC* (*Aeromonadaceae*) encoding a phosphate ABC transporter. These horizontal core gene families presented *Vibrionaceae*/*Aeromonadaceae*-like properties; therefore, HGTs occurring in the core gene families led to the misclassification of this taxon in the past.

10.1128/mSystems.00448-20.8 TABLE S5 List of core gene families acquired by HGT. Download Table S5, XLSX file, 0.02 MB. Copyright © 2020 Yin et al. 2020 Yin et al. This content is distributed under the terms of the Creative Commons Attribution 4.0 International license.

The nonsynonymous (*dN*) to synonymous (*dS*) substitution rates (*ω*) and positively selected sites were estimated for each horizontal core gene families to investigate the conservation and evolutionary pressure of these families. Based on the model 0 (M0 [one-ratio]) model, all 130 horizontal core gene families were confirmed to have undergone purifying selection, with an average *ω* value of 0.036 ± 0.05 (Table S5). This result revealed that purifying selection is a predominant action within horizontal core gene families and has contributed to the conservation of function in P. shigelloides. Although the whole coding regions were affected by the purifying selection, we identified numerous codon sites within the horizontal gene families that were subjected to positive selection. On the basis of a more stringent model (M8), we found that 64 horizontal core gene families contained codon sites which underwent positive selection, with an average *ω* value of 2.170 ± 1.31. Each gene family contained an average of 3.047 ± 3.17 positively selected sites. Our results suggested that the presence of adaptive evolution represents adaptation to diverse niches and rapid functional diversification in these horizontal core gene families.

### Numerous mobile genetic elements mediated the genomic plasticity.

**FIG 4:**
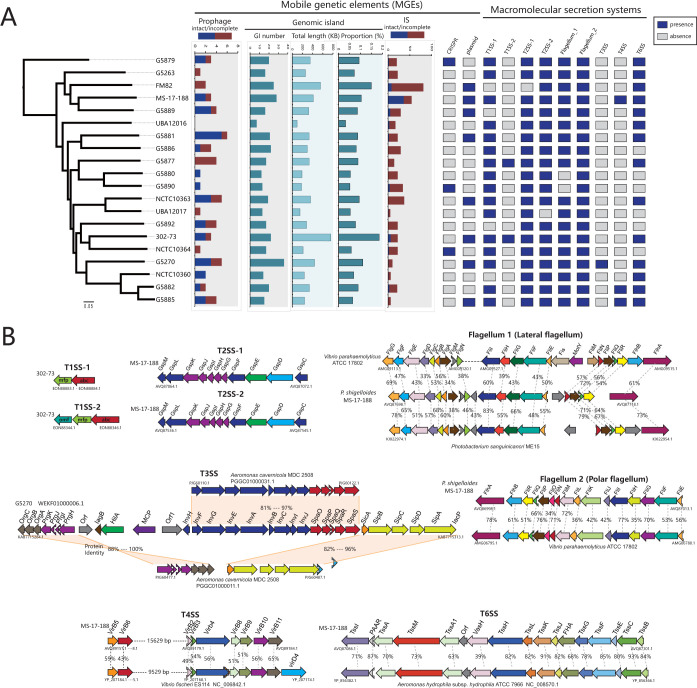
Mobile genetic elements (MGEs) and macromolecular secretion systems in P. shigelloides. (A) The distribution of MGEs and macromolecular secretion systems. (B) The genetic organization of macromolecular secretion systems. Identical genes are shown in the same color and linked by dotted lines. The percentages of protein identities of all homologous genes are shown. Due to the fragmentation of the draft genome, the T3SSs of *A. cavernicola* MDC 2508 were located in two contigs (PGGC01000011.1 and PGGC01000031.1).

Mobile genetic elements (MGEs) can mediate DNA acquisition and facilitate transmission of genetic material between different bacterial taxa (11). We detected multiple types of MGEs in the P. shigelloides genome, including prophages, genomic islands, insertion sequences (ISs), clustered regularly interspaced short palindromic repeats (CRISPRs), and plasmids (Fig. 4A; see also Table S6). These MGEs display a large and heterogeneous distribution model and could be a major driver of HGT and adaptive evolution of P. shigelloides. On average, one genome contained 2.8 ± 1.6 prophages and 24.8 ± 17.1 ISs. Overall, 24 types of the most homologous prophages were identified (Table S6), 3 of which were Vibrio_VP882, Vibrio_12B12, and Aeromo_phiO18P from *Vibrionaceae* and *Aeromonadaceae*. A genome harbored an average of 18.5 ± 6.2 genomic islands that were 317.8 ± 147.6 kb in size (8.5% ± 3.5% per genome) (Fig. 4A). The genomic island genes are detailed in Table S6 and were mainly enriched in the categories of “K: transcription,” “L: replication, recombination, and repair,” “M: cell wall/membrane/envelope biogenesis,” and “X: mobilome” (Fig. S2B and C). Furthermore, six types of plasmid elements were identified in eight strains (FM82, MS-17-188, G5889, G5881, NCTC10363, 302-73, G5270, and G5885) (Fig. 4A; see also Table S6). It was reported previously that plasmids may contribute to the multidrug resistance of P. shigelloides (17). Due to the limitations of the draft genome sequences, we could not perform a more detailed analysis of the plasmids in this study.

10.1128/mSystems.00448-20.9 TABLE S6 List of prophages, genomic islands, plasmids, and virulence factors in the VFDB and of resistance genes in the CARD database identified in P. shigelloides genomes. Download Table S6, XLSX file, 0.3 MB. Copyright © 2020 Yin et al. 2020 Yin et al. This content is distributed under the terms of the Creative Commons Attribution 4.0 International license.

Three strains possessed two types of CRISPR, including type I-F (G5879 and G5890) and type I-E (NCTC10364) (Fig. S3). We identified the putative homologous CRISPR-Cas system using a blastp search of the NCBI nonredundant protein database. We found that the CRISPR-Cas system of Vibrio cholerae HC-36A1 was most homologous to the type I-F CRISPR-Cas system of G5879 and G5890 and that the CRISPR-Cas system of Aeromonas caviae T25-39 exhibited the highest homology to the type I-E CRISPR-Cas system of NCTC10364 and that they also exhibited similar organizations of gene loci and presented identities of approximately 90% and 86% between protein sequences, respectively (Fig. S3). Additionally, they exhibited distinct spacer contents and organizations (Fig. S3). Generally, there are numerous MGEs in P. shigelloides that have contributed to its genomic plasticity. Our results also exhibit instances of MGE transmission between P. shigelloides and the predominant donor taxa, *Vibrionaceae* and *Aeromonadaceae.*

10.1128/mSystems.00448-20.3 FIG S3 Structures of CRISPR-Cas systems in P. shigelloides. Download FIG S3, EPS file, 1.8 MB. Copyright © 2020 Yin et al. 2020 Yin et al. This content is distributed under the terms of the Creative Commons Attribution 4.0 International license.

### Macromolecular secretion systems reflected the pathogenic potential of P. shigelloides.

Proteins secreted by bacteria are involved in many important tasks such as nutrient acquisition, adaptation to different niches, antibiotic resistance, and virulence (30, 31). In particular, many virulence factors in pathogens are secreted (32). Here, we revealed the occurrence of macromolecular secretion systems in 20 P. shigelloides genomes. The distributions and organizations of the macromolecular secretion systems represented in Fig. 4 (type one secretion system 1 [T1SS-1], T2SS-1, T2SS-2, Flagellum 1, Flagellum 2, and T6SS) were prevalent in P. shigelloides, and T1SS-2, T3SS, and T4SS exhibited sporadic distribution patterns (Fig. 4A). These secretion systems play roles in bacterial genome plasticity and pathogenicity. T1SS could secrete many proteins related to pathogenesis, nutrient acquisition, and antibacterial activity (33, 34). The following two types of T1SS were identified in P. shigelloides genomes: T1SS-1 occurred in 16 of the 20 strains, and T1SS-2 was present in only 2 strains. Previous studies revealed that T2SS is essential for virulence factor secretions and gut colonization (35, 36). We also found that two types of T2SS were widely distributed in P. shigelloides genomes, which indicates that the presence of T2SS may represent a general property for P. shigelloides (Fig. 4).

The pan-genome of P. shigelloides contained two gene clusters coding two types of flagellum systems, including a putative lateral flagellum gene cluster (designed Flagellum 1) and a putative polar flagellum gene cluster (designed Flagellum 2) (Fig. 4B). Two strains (G5880 and G5890) lacked Flagellum 1; however, Flagellum 1 and Flagellum 2 were present in the rest of the strains. Both flagellum gene clusters were previously identified as representing potential horizontal genes acquired from *Vibrionaceae* (Table S4). The lateral flagellum loci of Photobacterium sanguinicancri ME15 were most homologous to Flagellum 1, and the polar flagellum loci of Vibrio parahaemolyticus ATCC 17802 exhibited the highest homology to Flagellum 2. We also compared Flagellum 1 to its homolog in the potential donor taxon *Vibrionaceae* (Fig. 4B). The organizations of Flagellum 1 and Flagellum 2 were almost identical to those of the homologous loci in Vibrio parahaemolyticus ATCC 17802. Flagellar motility is one of the most pressure-sensitive cellular processes present in aquatic bacteria (37); therefore, acquisition of the flagellum system from cohabitating bacteria (such as *Vibrionaceae*) via HGT appeared to represent an ecological adaptation of P. shigelloides for functionality in aquatic environments. Similarly, *Photobacterium* spp. also acquired flagellar systems via HGT for swimming under deep-sea conditions (37, 38). Furthermore, lateral flagella and polar flagella of *Vibrio* spp. have also been reported to be essential for pathogenicity, being implicated in such activities as colonization, chemotaxis, host cell adherence, and invasion (39, 40). Thus, these two flagellum systems in P. shigelloides might play important roles in its pathogenicity.

One strain, G5270, had a complete set of T3SS genes (Fig. 4B). The set of genes was most homologous to that of Aeromonas cavernicola MDC 2508, with 81% to 100% identity between protein sequences. Although they exhibited similar organizations of gene loci, they had a high proportion of rearrangement. T3SS contributed to intracellular pathogenesis by direct transmission of proteins from the bacterial cytosol into the host cells (41, 42). It is worth noting that the G5270 strain with T3SS has the potential to cause an invasive disease. Three genomes (MS-17-188, G5882, and G5885) possessed type T-T4SS in its plasmid, which is a conjugation-related T4SS. The T4SS of Vibrio fischeri ES114 was closely related to the T4SS of P. shigelloides (Fig. 4B). Conjugation-related T4SS might mediate contact-dependent DNA transmissions and promote genetic exchange in P. shigelloides (43). The T6SS is widely present in Gram-negative bacteria and plays a critical role in the virulence and fitness of bacteria within a specific niche (44). In our study, 14 genomes had a complete set of T6SS genes. The T6SS of Aeromonas hydrophila subsp. *hydrophila* ATCC 7966 exhibited the highest homology to P. shigelloides. The two species exhibited similar organizations of gene loci and showed approximately 83% identity between protein sequences (Fig. 4B). T6SS among *Aeromonas* spp. has been reported to operate as a form of phage tail-spike-like injection machinery that translocates virulence factors directly into the host cell’s cytoplasm (45, 46), suggesting the potential for cytotoxicity and extraintestinal infection of P. shigelloides. Future studies are required to confirm the function of these genetic elements in P. shigelloides and their potential role in pathogenicity.

### Virulence and antimicrobial genotypic and phenotypic profiles in P. shigelloides.

**FIG 5:**
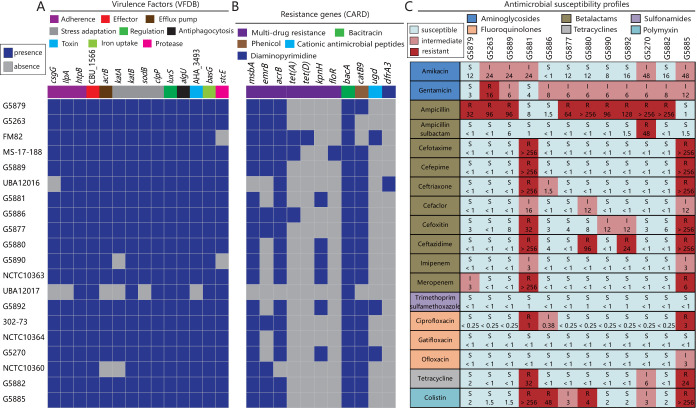
The genotypic and phenotypic profiles of virulence factors and resistance genes across all 20 P. shigelloides genomes. Blue coloring represents the presence of a gene, and gray represents absence. (A) Heat map of the distribution of virulence factors. For the current presentation, we removed the virulence factors of the previous macromolecular section systems and O-antigen/LPS/capsule. (B) Heat map of resistance gene distribution. (C) Antimicrobial susceptibility profiles of P. shigelloides.

All P. shigelloides genomes were locally compared against the Virulence Factors Database (VFDB) to detect virulence genes. The distribution of virulence genes among the strains is summarized in Fig. 5A and Table S6. For the current presentation of Fig. 5A, we removed the virulence factors of the previous macromolecular section systems and the O-antigen/lipopolysaccharide (LPS)/capsule. We found 14 virulence factors in the 20 P. shigelloides genomes, including adherence (*csgG*, *ilpA*, and *htpB*), effector (CBU_1566), efflux pump (*acrB*), stress adaptation (*katA*, *katB*, *sodB*, and *clpP*), regulation (*luxS*), antiphagocytosis (*algU*), toxin (AHA_3493), iron uptake (*basG*), and protease (*stcE*) virulence factors. Most of these virulence factors were prevalent in these strains (Fig. 5A), suggesting they might play important roles in pathogenicity. Six virulence factors were identified as being encoded by core genes (*htpB*, CBU_1566, *katB*, *clpP*, *luxS*, and *basG*) shared by all 20 strains. Three virulence factor genes, *acrB*, *katA*, and *katB*, were previously identified as potential horizontal genes, emphasizing the importance of HGT’s role in pathogenicity.

Previous studies found that P. shigelloides is generally susceptible to most available antibiotics (15, 16); however, singly or multiply drug-resistant strains have also been reported (17). Here, we detected the dynamics of resistance genes in P. shigelloides. Eleven resistance genes associated with five different classes were identified (Fig. 5B). As shown in Fig. 5B, *msbA*, *acrB*, *bacA*, and *catB9* were prevalent in these strains, and the other genes exhibited sporadic distribution patterns. The resistance mechanisms of these resistance genes include efflux, target alteration, inactivation, and replacement of antibiotics (Table S6). Most of the strains contained multiple resistance genes related to multidrug resistance, including resistance to bacitracin, phenicol, cationic antimicrobial peptides, and diaminopyrimidine. The antimicrobial genotypic characteristics described for these strains differ, and no robust correlation was established between the genotypic profile and phylogenetic origin, suggesting that sporadic HGT might be the major driver of resistance gene acquisition.

All 12 of our P. shigelloides strains were tested for susceptibility against 18 antibiotics, including aminoglycosides, beta-lactams, sulfonamides, fluoroquinolones, tetracyclines, and polymyxin. As shown in Fig. 5C, all of the strains were uniformly susceptible to trimethoprim-sulfamethoxazole and gatifloxacin. The majority of the strains were susceptible to amikacin, ampicillin-sulbactam, cefotaxime, cefepime, ceftriaxone, cefaclor, cefoxitin, ceftazidime, imipenem, meropenem, ciprofloxacin, ofloxacin, tetracycline, and colistin. The greatest resistance to antibiotics was noted with ampicillin; however, ampicillin combined with sulbactam was able to greatly inhibit the growth of most strains. The majority of the strains showed intermediate susceptibility to gentamicin. Notably, two strains, G5881 and G5885, showed multidrug resistance to cefotaxime, cefepime, ceftriaxone, cefoxitin, ceftazidime, meropenem, ciprofloxacin, tetracycline, and colistin. However, we did not detect any more resistance genes in the G5881 and G5885 strains (Fig. 5B). Abdelhamed et al. reported that multidrug-resistant strain MS-17-188 possessed three plasmids which were found to carry several resistance genes (17), suggesting that plasmid-mediated HGT might play an important role in the antimicrobial susceptibility of P. shigelloides. Thus, the observation of fewer resistance genes in the G5881 and G5885 strains might have been a result of incompleteness of the draft genome. Overall, most of 12 P. shigelloides strains were susceptible to most of the antibiotics tested in this study; the antimicrobial susceptibility of these strains was heterogeneous. The risk of multidrug resistance deserves further attention.

### Conclusion.

This study evaluated the taxonomic position, evolutionary dynamics, and pathogenicity of P. shigelloides based on core genome phylogeny and on pan-genome and comparative genomic analysis, which provided us with a comprehensive understanding of the genomic perspective of P. shigelloides. The core genome phylogeny, Neighbor-Net network, and comparison of ANI data revealed a clear distinction between P. shigelloides and other members of *Enterobacterales*. The open pan-genome of P. shigelloides presented extensive genetic diversity and a large and flexible gene repertoire. The low clonality of the core genome phylogeny indicated that frequent recombination had occurred for the current niche of each strain. The core and accessory genomes have distinct evolutionary histories, suggesting that the diverse accessory genomes accounted for an important proportion of the evolution of P. shigelloides. The large number of gene expansions and contractions exhibited high levels of genomic plasticity, which shaped the divergence of functions among the P. shigelloides strains. The massive gene contraction suggested that gene loss might play an important role in the adaptive evolution of P. shigelloides. We concluded that the genetic diversity of P. shigelloides was most significantly derived from HGT and that many transfers were related to biochemical properties and pathogenicity. HGT conferred a novel gene repertoire from diverse donor taxa. We have presented massive HGT from *Vibrionaceae* and *Aeromonadaceae*, which led to the historical taxonomic confusion and conferred donor-like properties, including those corresponding to the member transporter, energy metabolism, motility, pathogenicity, and antimicrobial resistance. We found that all horizontal core gene families tended to undergo purifying selection, which is the main force acting on the evolution of the horizontal core gene families in P. shigelloides. In addition, approximately half of horizontal core gene families contained positively selected sites, which were found to be associated with differential responses to the environmental changes. The presence of numerous MGEs that included prophages, genomic islands, ISs, and CRISPRs may promote the apparently high rates of HGT.

P. shigelloides also presented the potential for intestinal and invasive pathogenesis characterized by T1SS, T2SS, T3SS, T4SS, T6SS, Flagellum 1, Flagellum 2, virulence factors, and resistance genes. It is worth noting that P. shigelloides appeared to have acquired Flagellum 1, Flagellum 2, and T4SS from *Vibrionaceae* and T3SS and T6SS from *Aeromonadaceae*, indicating that virulence gene acquisition was important for the pathogenesis of P. shigelloides. Through antimicrobial susceptibility testing with 18 antibiotics, we were able to determine that most strains were resistant to ampicillin and gentamicin but were susceptible to other antibiotics. Two strains were resistant to multiple antibiotics, emphasizing the risk of multidrug resistance. Our genome data and results provide valuable information to enable better understanding of the phylogenetic position, evolutionary dynamic, and pathogenic potential of P. shigelloides.

## MATERIALS AND METHODS

### Data collection.

In this study, we sequenced draft genomes of 12 P. shigelloides strains, all of which have been found to have different lipopolysaccharides. All of the strains were cultured in Tryptone soya broth (TSB) (QingDao ShuiRi Bio-Technologies Co., Ltd., Qingdao, China) at 37°C overnight with shaking.

### Genome sequencing and row data processing.

DNA was prepared from 1 ml of cultures grown overnight with a Wizard genomic DNA purification kit (Promega) according to the manufacturer’s instructions. The genomic sequencing was performed using Solexa pair-end sequencing technology (Illumina, Little Chesterford, Essex, United Kingdom), with a depth of 90-fold to 100-fold coverage. The reads were subjected to *de novo* assembly using VelvetOptimiser v2.2 (47). The annotation of newly sequenced genomes was performed using the NCBI Prokaryotic Genome Annotation Pipeline (https://www.ncbi.nlm.nih.gov/genome/annotation_prok).

### Phylogenetic analysis based on core genome and pan-genome.

Orthologous groups were delimited using OrthoFinder (48), in which all the protein sequences were compared using a BLASTp all-against-all search with an E value cutoff of 1e−3. The single-copy core gene families, core gene families, and pan-gene families were extracted from the OrthoFinder output files. Nucleotide sequences of the single-copy core gene families were extracted according to the accession numbers of the proteins and then aligned using MAFFT (49). Phylogenetic analysis (Fig. 1A) of 79 genomes was performed using the set of single-nucleotide polymorphisms (SNPs) present in 307 single-copy core gene families (see Table S7 in the supplemental material). The SNPs were integrated according to the arrangement of the genes on the MS-17-188 complete genome. The phylogenetic analysis of P. shigelloides was performed using the set of SNPs present in 1,522 single-copy core gene families (Table S7). The SNPs were also integrated according to the arrangement of the genes on the MS-17-188 complete genome. It was predicted that homologous recombination could occur in bacterial genomes and might confound the phylogenetic analysis. We identified and removed the putative recombinational regions of SNPs, using ClonalFrameML software (50). The maximum likelihood (ML) tree was constructed using MEGA 7 software (51) (with the General Time Reversible [GTR] model). Phylogenetic analysis of *Enterobacterales* was performed using 307 single-copy gene families shared by 79 *Enterobacterales* strains. The Neighbor-Net network was constructed and visualized with SplitsTree4 (52) with uncorrected p-distance transformation.

10.1128/mSystems.00448-20.10 TABLE S7 List of single-copy core gene families identified in 79 genomes and 20 P. shigelloides genomes. Download Table S7, XLSX file, 0.1 MB. Copyright © 2020 Yin et al. 2020 Yin et al. This content is distributed under the terms of the Creative Commons Attribution 4.0 International license.

The pan-genome phylogenetic analysis was performed based on the absence or presence of each gene family among 6,056 pan-gene families using the Manhattan distance to measure the evolutionary relationships of strains. A pan-genome tree based on this Manhattan distance matrix was constructed using MEGA 7 with the neighbor-joining (NJ) method. The congruence between the core genome tree and the pan-genome tree was evaluated by calculating normalized Robinson-Foulds (nRF) and normalized matching-cluster (nMC) scores using TreeCmp (53). A comparison of the core genome tree and the pan-genome tree was constructed using the Dendroscope 3 program (54).

### Core and pan-genome analysis.

The pan-genome analysis was performed using Heap’s law for pan-genome models as described in a previous study (24). The total number of gene families for increasing values of the number of genomes (*N*) is shown. The curve represented a least-squares fit (based on the power law [*n* = ${кN}^{\gamma }$]) to the averages. The regression analysis for the core gene family curve was performed using a weighted least-square regression by fitting the power law *n* =*к*exp(*m* ×N)+*Θ* to means (55), where *N* represents the number of genomes, *n* represents the number of core gene families, *Θ* is a constant value representing the predicted minimum number of core genes, and *к* and *m* are parameters.

### Gene expansion and contraction.

To gain greater insight into the evolutionary dynamics of the gene families, representations of the expansion and contraction of the gene families in each branch among the 20 P. shigelloides strains were constructed using CAFÉ (v3.1) (56) with default parameters.

### Identification of potential horizontal genes.

A total of 20 P. shigelloides genomes were analyzed for the presence of horizontal genes using HGTector (57) software with BLASTp parameter thresholds of 60% identity and 60% coverage and an E value of 1e−6. *Plesiomonas* (rank, genus; taxon identifier [ID], 702) and *Enterobacterales* (rank, order; taxon ID, 91347) were set as self-group and close group, respectively. The horizontal genes among P. shigelloides genomes and potential donors were identified and extracted from the HGTector output files.

### Selective pressure analysis.

Representations of results of the evolutionary pressure analysis were constructed by the use of horizontal core gene families and EasycodeML(58). The core genome tree (Fig. 2C) was applied to analysis. Positive selection in gene families can be estimated by calculating the ratio of the nonsynonymous substitution rate to the synonymous substitution rate (*dN*/*dS*, represented by the *ω* parameter in Codeml/EasycodeML). To test for positive selection at different codon sites, we estimated parameters under three different codon substitution models (M0 [one-ratio model], M7 [beta model], and M8 [beta plus ω > 1]), and representations of the results of the comparison (M7 versus M8) were constructed using likelihood ratio tests (LRTs) with the chi-square model in EasycodeML (58). The value of the *ω* parameter and positively selected sites of horizontal core gene families are presented in Table S5.

### Comparative genomic analysis.

The average nucleotide identity (ANI) and amino acid identity (AAI) values were calculated (Fig. 1B) using JSpecies 1.2.1 software (19) and CompareM software (https://github.com/dparks1134/CompareM), respectively. We analyzed the functional category of the gene family using the Cluster of Orthologous Groups (COG) assignment. The functional annotation of proteins was performed by alignment against the COG database of NCBI using BLASTp with an E value of 1e−5. The PHAge Search Tool Enhanced Release (PHASTER) was utilized to find the prophages (59). Genomic islands were predicted using the IslandViewer 4 database (60). Insertion sequences (ISs) were predicted using the ISFinder database (61). Clustered regularly interspaced short palindromic repeats (CRISPRs) were predicted using the CRISPR recognition tool (CRT1.2) with default parameters (62). Plasmid detection was performed by using PlasmidFinder (63).

### Identification of virulence factors and resistance genes.

To identity the virulence factors and resistance genes, the protein sequences of all genomes were aligned using BLASTp with an E value cutoff value of <1e−6, an identity value of >60%, and a coverage value of >60% against the data set from Virulence Factors Database (VFDB) (64) and the Comprehensive Antibiotic Database (CARD) (65). To examine the virulence-related elements, we screened gene clusters using the large-scale blast score ratio (LS-BSR) tool (66). The results were visualized using pheatmap R packages.

### Antimicrobial susceptibility testing.

Antimicrobial susceptibility testing was performed for 18 antibiotics, including ceftriaxone, cefaclor, cefoxitin, ceftazidime, cefotaxime, cefepime, colistin, ciprofloxacin, gentamicin, gatifloxacin, meropenem, trimethoprim-sulfamethoxazole, ofloxacin, ampicillin, imipenem, amikacin, tetracycline, and sulbactam. MICs were determined by the broth microdilution using AST-N334 and AST-N335 dehydrated panels (bioMérieux, Vitek2) according to standard protocols. Resistance was defined using CLSI criteria.

### Data availability.

The sequence data determined in this work are available in the NCBI GenBank database under project accession no. PRJNA576348. The background information and accession numbers for the strains used in this study are presented in Table S1.
